# Self-management with alcohol over lifespan: psychological mechanisms, neurobiological underpinnings, and risk assessment

**DOI:** 10.1038/s41380-023-02074-3

**Published:** 2023-04-28

**Authors:** Christian P. Müller, Gunter Schumann, Jürgen Rehm, Johannes Kornhuber, Bernd Lenz

**Affiliations:** 1 Department of Psychiatry and Psychotherapy, University Hospital, Friedrich-Alexander-University Erlangen-Nürnberg, Schwabachanlage 6, 91054 Erlangen, Germany; 2 Centre for Drug Research, Universiti Sains Malaysia, 11800 Minden, Penang Malaysia; 3 Institute of Psychopharmacology, Central Institute of Mental Health, Medical Faculty Mannheim, Heidelberg University, Mannheim, Germany; 4 The Centre for Population Neuroscience and Stratified Medicine (PONS), ISTBI, Fudan University, Shanghai, China; 5 PONS Centre, Charite Mental Health, Department of Psychiatry and Psychotherapie, CCM, Charite Universitaetsmedizin Berlin, Berlin, Germany; 6 Institute for Mental Health Policy Research, Centre for Addiction and Mental Health, Toronto, ON M5S 2S1 Canada; 7 Dalla Lana School of Public Health, University of Toronto, Toronto, ON M5T 3M7 Canada; 8 Department of Psychiatry, University of Toronto, Toronto, ON M5T 1R8 Canada; 9 Center for Interdisciplinary Addiction Research (ZIS), Department of Psychiatry and Psychotherapy, University Medical Center Hamburg-Eppendorf (UKE), Martinistraße 52, 20246 Hamburg, Germany; 10 Department of Addictive Behavior and Addiction Medicine, Central Institute of Mental Health, Medical Faculty Mannheim, Heidelberg University, J5, 68159 Mannheim, Germany

**Keywords:** Addiction, Molecular biology

## Abstract

Self-management includes all behavioural measures and cognitive activities aimed at coping with challenges arising throughout the lifespan. While virtually all of these challenges can be met without pharmacological means, alcohol consumption has long been instrumentalized as a supporting tool to help coping with problems arising selectively at adolescence, adulthood, and ageing. Here, we present, to our knowledge, the first systematic review of alcohol instrumentalization throughout lifespan. We searched MEDLINE, Google Scholar, PsycINFO and CINAHL (from Jan, 1990, to Dec, 2022) and analysed consumption patterns, goals and potential neurobiological mechanisms. Evidence shows a regular non-addictive use of alcohol to self-manage developmental issues during adolescence, adulthood, and ageing. Alcohol is selectively used to overcome problems arising from dysfunctional personality traits, which manifest in adolescence. A large range of psychiatric disorders gives rise to alcohol use for the self-management of distinct symptoms starting mainly in adulthood. We identify those neuropharmacological effects of alcohol that selectively serve self-management under specific conditions. Finally, we discuss the adverse effects and associated risks that arise from the use of alcohol for self-management. Even well-controlled alcohol use adversely impacts health. Based on these findings, we suggest the implementation of an entirely new view. Health policy action may actively embrace both sides of the phenomenon through a *personalized informed use* that allows for harm-controlled self-management with alcohol.

## Introduction

Alcohol is one of the oldest psychoactive drugs and is deeply rooted in human civilisation. During the last few thousand years, it has penetrated many cultures of the world [1–4]. It is currently the most frequently used psychoactive drug in the world. Its production and distribution are significant economic factors. In western societies, alcohol is strongly connected with many routines of daily life [5].

Alcohol may occur naturally in the reproductive structures of many angiosperm plants, or it can be produced by fermenting nectar or ripe fruit [6]. As such, it is naturally available and many species have been exposed to it for a long time throughout evolution [7]. The consumption of fermentation-generated alcohol emerged as a behaviour early in evolution, which entails long periods for metabolic adaptations and genetic inheritance of physiological traits dealing with alcohol. Active alcohol consumption was observed already in fruit flies [8], rodents [6, 9], monkeys [6], and many other species [7] when they had access to naturally occurring alcohol. Many systematic studies in animals have shown that they can learn to seek and consume alcohol and work for its availability [10]. Based on these observations, it was suggested that the behavioural capacity to seek and consume alcohol is not restricted to humans, but emerged much earlier in evolution [3, 4]. The capability to instrumentalize the effects of plant-derived psychoactive drugs is known from animals [11, 12]. Drug consumption for the self-medication of infections, gastrointestinal problems, and other physically adverse conditions is reported [12] as a learned, inherited behaviour [4, 13]. Thus, the consumption and instrumental use of alcohol have an evolutionary origin, starting long before human beings emerged. The occurrence of genetic variants in the enzyme alcohol dehydrogenase (ADH) in hominids, which enhanced the metabolization of alcohol in, e.g., fermenting fruits [14], enabeled increased alcohol consumption. Part of this evolutionary history and beneficial alcohol use is the human understanding that alcohol kills germs, and therefore, represents a safer and healthier alternative to available water sources. Deliberately using alcohol to change mental states at dedicated times and on special occasions has long been recognized as a driver of human social and cultural evolution [15, 16].

Unlike our prehistoric ancestors, virtually all modern humans are aware that alcohol is toxic and can cause severe damage to the individual and their environment. Alcohol use causes addiction in a small percentage, but in total a very considerable number, of consumers [17]. The main scientific paradigm of the last decades has been, therefore, to view alcohol as a pharmacological reward [18] and addictive drug [19] with accepted, but in its essence, only negative consequences.

Human beings are engaged in permanent ‘self-management’, which adjusts physical well-being and interaction with the environment. There are two major ways humans react when homeostasis is threatened. Firstly, one may change the perception and cognitive interpretation of the self in the environment. The second powerful way is to change goal-directed behaviour. This includes the use of tools that increase the efficacy of a behaviour, i.e., a goal can be reached with less effort or more accurately [5]. A specific class of tools or instruments that humans exploit for both self-management strategies are psychoactive drugs. These are chemical compounds that change physiological and neurobiological function of the organism and/or interact with cognition and impact the efficacy of goal-directed behaviour. The most widely instrumentalized drug for self-management is alcohol (ethanol) [3–5,20–22]. However, human beings with a genetic risk or submitted to environmental risk conditions can develop alcohol use disorder (AUD) [23–28].

Even chronic alcohol consumption does not lead to addiction in the vast majority of users. The US National Survey on Drug Use and Health (NSDUH 2021) revealed that 47.5% of Americans aged 12 or older reported current (past month) alcohol use [29]. European surveys revealed that in the general adult population of 15 to 64-year-olds, up to 77% per country showed current (last month) alcohol consumption [30]. Of those who admitted to being current alcohol drinkers in the US, less than half were binge drinkers (45%), and ‘only’ about 12% reported heavy alcohol use [29]. In the EU, 37% of adolescents (aged 15–16) and 19% of adults (aged 18+) reported heavy episodic drinking at least once a month [31]. This may support the view that alcohol consumption in non-addicted individuals is not driven by alcohol’s pharmacological reinforcing action and an initiated addiction trajectory that would eventually lead every user to AUD. Instead, it may be maintained by evolutionary developed benefits that may frequently outweigh the possible harm [3, 4]. Understanding these processes may not only explain the often highly sophisticated mechanisms of consumption, but also improve AUD prevention and treatment. Although reports of its potential benefits and instrumental alcohol use have been accumulating for decades now, no evidence-based overview has been developed. Therefore, here we review the psychological mechanisms of how alcohol is used for self-management along the lifespan in a systematic way. We outline neurobiological mechanisms that support beneficial effects and discuss selective harm potentials.

## Search strategy and selection criteria

We searched MEDLINE, Google Scholar, PsycINFO and CINAHL for relevant, high-quality studies published in English. Those included empirical data reports, epidemiological studies, cohort studies and population surveys published from Jan 01 1990 to Dec 31 2022. Sources also included commonly cited and highly regarded older publications. Search terms used were “alcohol”, “alcohol use”, “alcohol abuse”, “alcohol addiction”, “alcohol dependence”, alone and together with “self-management”, “drug/alcohol instrumentalization”, “epidemiology”, “development”, “adolescence”, “adulthood”, “old age/ageing”, “personality traits” and “psychiatric disorders”. Furthermore, reference lists from articles identified in this way were searched and analysed when relevant. This search yielded 517 references across the theme that were grouped systematically according to self-management goals along lifetime. While goals were identified and described as completely as possible, qualitative literature does not allow for quantifiying user numbers or alcohol use effect sizes. Respective references are provided here in an exemplary way.

## Alcohol for self-management during the lifespan

**Fig. 1:**
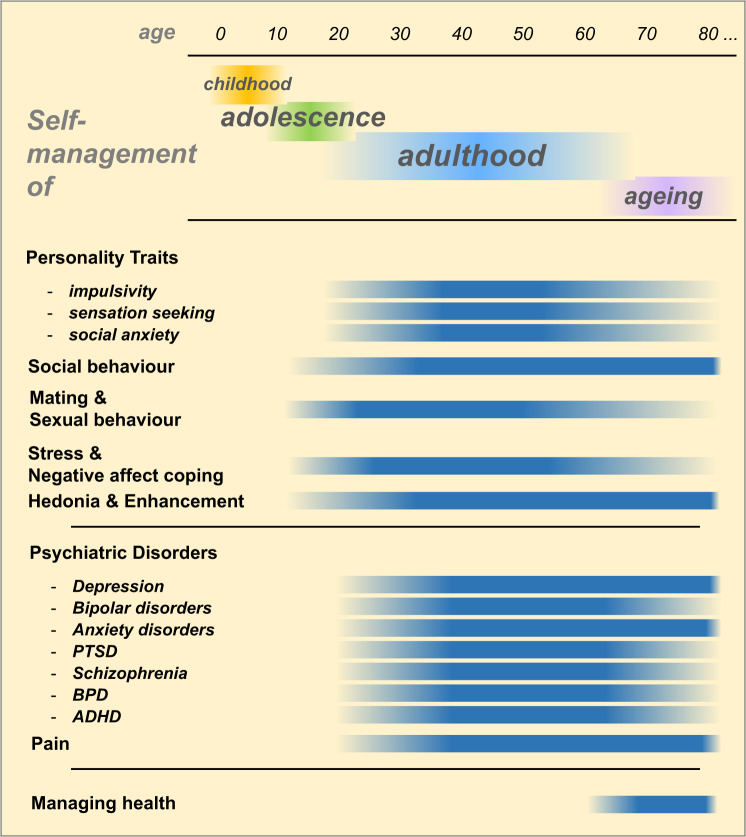
Self-management with alcohol over the life span. Onset and duration for reported beneficial effects of non-addicted alcohol consumption (ADHD attention deficit hyperactivity disorder, BPD borderline personality disorder, PTSD posttraumatic stress disorder).

The human body and mind are submitted to life-long development and changes. Each developmental period comes with unique tasks and challenges for the individual. Self-management tasks, an extension and broadening of the self-regulation already developed in childhood [32, 33], appear and disappear during these periods. Below we discuss environmental challenges specific to developmental periods throughout the lifespan during which alcohol is consumed (Fig. 1).

### Adolescence

Adolescence is the period from the end of childhood to adulthood. It coincides roughly with puberty and, according to the World Health Organization (WHO) definition, with an age from 10 to 19 years. Occasionally, studies consider the age from 19 to 24 as late adolescence. This period involves profound body growth and brain maturation. Individuals reach physical and mental independence from their parents and reproductive fitness [34, 35]. Depending on the first subjective drug effects [36, 37], an initially undifferentiated consumption may develop into a highly specific pattern of alcohol use [38, 39]. In adolescents two main categories dominate drinking motives: namely the belief that alcohol will enhance positive affect (enhancement) or the belief that alcohol will help cope with negative affect (coping). Each motive can be further divided into self-focused or externally focused [40–42]. These self-management motives [43], which closely mirror the previously suggested instrumentalization goals [3–5], have been confirmed by a plethora of studies on adolescents and young adults from different backgrounds [44–46]. However, there is also a motive of ‘not to drink’, which explicitly limits consumption in adolescents [47].

#### Alcohol in the self-management of personality traits

Human beings show a well-known variance in their personality traits, which strongly manifest in adolescence and remain during later periods of life. The expression of these traits may cause problems when they deviate too much from population norms. These problems manifest at a behavioural level, when inappropriate behaviours cause negative consequences and at a subjective level when emotion, motivation and cognition cause adverse mental states [38, 48].

##### Impulsivity

Impulsivity, as part of common behaviour, is the predisposition for rapid, insufficiently planned responses to internal and external stimuli without full appreciation of potential negative consequences. As such, impulsive actions may provide advantages in situations of restricted time and high decision pressure using less than full capacity for decision-making. This may, under situations of more time and cognitive resources, however, yield disadvantageous outcomes. Several dimensions of impulsivity have been defined according to their distinct assessment and related neuronal substrates: self-rated trait impulsivity, impulsive choice and impulsive behaviour [49]. Impulsivity as a personality trait is characterized by reduced reflectiveness, rapid decision-making and action and a failure to inhibit behaviour [50]. Impulsivity may result in externalizing behaviour and conduct problems [51, 52]. High trait impulsivity is associated with the early onset of alcohol consumption during adolescence and enhanced alcohol use during adulthood [53, 54]. In particular, the relapse risk is then predicted by high impulsivity in patients with AUD [55]. However, alcohol consumption and AUD development may, by its effects on prefrontal brain volume [56], enhance trait impulsivity and reduce behavioural inhibition [57], making this relationship bidirectional [58] (Fig. 1).

##### Sensation seeking

This personality trait is characterized by behavioural tendencies to seek novel, complex and intense sensations and experiences. There is a low tolerance for boredom and a strong need for stimulation [59]. This trait is largely congruent with the concept of “novelty seeking” [60, 61]. The sensation-seeking trait has been associated with increased alcohol use in otherwise healthy people and in patients with emerging psychiatric disorders in late adolescence and early adulthood [62, 63].

##### Social anxiety

As a personality trait, social anxiety arises in adolescence and persists through adulthood. When inappropriate, it can lead to social inhibition and avoidance of social interactions up to self-isolation. Individuals with a high level of social anxiety rather avoid social interactions or endure them with discomfort. There may be a persistent fear of criticism, rejection, defeat and feelings of humiliation [64]. Alcohol is used to reduce tension [65–67] and the physical signs of arousal in persons with high social anxiety [68, 69] in late adolescence and early adulthood. Predominantly individuals with a pronounced and unpleasant social stress response claim to benefit from alcohol consumption and its stress-reducing effects [70, 71]. Alcohol may decrease self-awareness and the emotional impact of negative self-evaluation after failure in social interactions in late adolescence and early adulthood [71, 72]. Alcohol limits perceptual capacity and restricts the processing of cues related to social threat and defeat, as shown for adults [71, 73, 74].

#### Social behaviour

Established alcohol beliefs and drinking with peers are major drinking motives for young people’s self-management of their position in a social group [75–77]. Moreover, a popularity goal may predict alcohol consumption in adolescents [78]. Inducing a comparative mental state among peers with alcohol [3] and engaging in synchronized behaviours with peers (e.g., having fun with friends, partying and celebrations) may enhance group bonding beyond drug-free interactions [79–81] as the most frequent alcohol use for self-management in adolescents [75].

#### Mating and sexual behaviour

Alcohol consumption can reduce inhibition when approach behaviour is initiated and romantic relationships are established in adolescence [82]. It can encourage the transition of peer preference to partner preference. It may reduce the conditioned inhibition of physical approach behaviour and enhance courage when sexual activity is initiated. Alcohol may also facilitate the acceptance of one’s own maturing body and the feeling of sexual arousal in adolescence [79].

#### Stress and negative affect coping

Negative mood control is a major self-management motive for alcohol consumption starting in adolescence and continuing throughout later life [3, 75, 83]. In particular, problem-focused thinking in a depressed and anxious mood appears to be associated with alcohol consumption for coping in late adolescence [84–86]. Besides a dampening of emotion and rumination around aversive themes, the exact mechanisms underlying this alcohol-induced effect are not yet understood.

#### Hedonia and enhancement

Alcohol has a dose-dependent effect on the hedonic tone. In low to medium doses, it produces mood enhancement, while at high doses, it can cause anhedonia and depression [9]. The exact doses cannot be clearly pointed out, as there is a large natural variance in the hedonic effects of alcohol between individuals. However, also a maximal alcohol-induced euphoria is relatively weak [87, 88]. Human beings self-titrate alcohol doses individually to induce mild hedonic effects. The enhancement motive is already established by the age of adolescence [89].

#### Problems with alcohol in adolescence

Already at adolescent age, self-management can go wrong. This can be conceived as an over-instrumentalization, i.e., when alcohol is used in increasing dose and frequency to self-manage new and persistent problems, but an effective dose window for alcohol is left [5, 90]. Chronic high-dose alcohol exposure, e.g., 7–9 standard drinks per day over an extended period of years [91], leads to changes in brain structure and function in a particularly vulnerable brain during adolescence. This does not only alter alcohol effects in self-management, but also impairs behavioural and cognitive flexibility in drug-free situations [92, 93]. High levels of consumption lead to organ damage in the developing organism and to disadvantageous behaviours, often involving increased risk-taking. At that stage, the toxic and harmful effects of alcohol dominate over the self-management benefits and eventually outweigh them [3–5,20]. Adolescents and individuals of all ages with this behavioural pattern show an enhanced risk of lethal intoxication, accidental injuries and suicide attempts.

In adolescents, drinking motives and perceived alcohol rewards were found to predict later alcohol consumption and alcohol abuse problems in late adolescents and early adults [94]. Social and coping motives were related to later alcohol misuse and AUD in mid and late adolescence and in early adulthood [95, 96]. Adolescent alcohol consumption is associated with an increased risk for AUD and other substance use disorders (SUD). It also enhances the risk for other mental disorders, such as conduct disorder, anxiety and depression. Alcohol-related problems and AUD can occur in mid to late adolescence [97]. Adolescent alcohol misuse and binge drinking may have adverse consequences in those behavioural domains for which alcohol is used, including academic problems, social problems, hangovers and risky sexual activity [98]. In addition, problems may also emerge in other domains, such as cognition, aggression, victimization, physical injuries (e.g., traffic accidents), emotional problems and suicidality [54]. Personality traits that are considered normal and do not cause adverse states, such as neuroticism, extraversion, openness to experience, agreeableness and conscientiousness, may well affect the psychological and neurobiological effects of alcohol and the risk of AUD development in late adolescence [83, 99]. Personality traits that were generally associated with AUD are sensation seeking, impulsivity, hopelessness, and anxiety sensitivity [100].

### Adulthood

Association studies have reported numerous benefits of moderate alcohol consumption during adulthood, such as more close friendships, and more family support than total abstinence [101, 102]. Compared to complete abstinence, moderate alcohol consumption is also associated with less anxiety and depression [101, 102] and better stress management. However, these studies do often not consider the *neuroeconomic trade-off* made by the individual consumer. For most, the brain makes unconscious intrinsic calculations that regularly update and compare the perceived benefits of the consumption against the mostly prospective and not yet fully encountered risks and side effects (opportunity costs). Thereby, the individual accepts many semantically known, but in its perception rather delayed hazards, like liver problems and reduced life expectancy, and also widely unknown risks, like increased cancer rate, in exchange for a proven, i.e., already perceived benefit.

#### Social behaviour

Moderate amounts of alcohol attenuate social inhibition and discomfort in social situations in adults in a similar way as in adolescents. Thereby, alcohol may reduce social anxiety and social performance anxiety and may enhance social approach behaviour [63, 101, 103]. Alcohol can increase perceived social bonding [104]. Non-addicts consume alcohol in, before, or during social gatherings to attenuate social anxiety [22, 44, 101]. Thereby, positive expectations of the social effects and self-efficacy moderate alcohol consumption in self-management [36, 76, 105]. Alcohol is also consumed to facilitate the management of friendships. In a social context, it allows for social behaviours that are rather suppressed under sober conditions, e.g., men talking about emotions [106] (Fig. 1).

#### Mating and sexual behaviour

Numerous studies support a strong relationship between alcohol consumption, mental state changes, and the perceived chances for sexual intercourse in adults [82]. Thereby, alcohol may exert favourable effects by temporarily overcoming conditioned fear in consensually approaching a potential partner at selected times (e.g., after work, weekend nights) and settings (e.g., bar, party).

#### Hedonia and enhancement

The enhancement motive, already established in adolescence [89], continues to be pursued in adulthood [22, 107].

#### Stress and negative affect coping

Consistent with adolescents, adults regularly report using alcohol to improve recovery and enhance coping with stress [22, 44, 101, 107, 108]. Challenges frequently imposed by environmental changes are reward devaluation and the extinction of previously rewarded behaviour. This can induce stress and even cause depression [109]. Alcohol is consumed to reduce tension caused by a highly stressful work environment [110], or from a partner relationship [111].

#### Alcohol in the self-management of psychiatric disorders

Certain psychiatric disorders coincide with enhanced alcohol consumption. This may start at late adolescence and gain full expression during adulthood. Affected individuals frequently report that they use alcohol to attenuate the symptoms and suffering from the disorders [112]. While alcohol may not be able to persistently restore homeostasis in mental disorders, it may, nevertheless, cause temporary relief from negative affective states and may support normal behaviour. In that, alcohol may temporarily enhance ‘functioning’ in everyday life [113]. However, it should also be noted that the use of alcohol may cause or aggravate psychiatric disorders, resulting in a comorbidity with AUD.

##### Depression

Depression is a mental disorder against which various pharmacological and non-pharmacological self-management measures are taken [114]. Alcohol consumption can provide temporal relief from negative affect and depressive symptoms [101, 115]. In particular, men who developed a ‘male depression’ reported self-managing this state with alcohol [116]. The self-management of depression with alcohol is often characterized by a refusal of medical treatment, with alcohol seen as a “quick solution” [116]. Epidemiological surveys revealed that about a quarter of patients with DSM-diagnosed mood disorders consume alcohol with the intention of managing their mood symptoms [117].

##### Bipolar disorders

Bipolar disorders are severe and chronic affective disorders. *Bipolar I disorder* is characterized by manic episodes, while Bipolar II disorder involves hypomanic episodes and a major depressive episode. There emerge recurring episodes of mood states with opposite polarity and transitory states. They include manic, hypomanic, euthymic and major depressive episodes with partially rapid cycling [118, 119]. Patients with bipolar disorders consume alcohol to effectively alleviate dysphoric moods, including boredom, sadness, or depression [117, 120]. They also report alcohol use during manic episodes for coping [117]. When in a positive mood, patients use alcohol to enhance euphoria [120, 121], and to be more sociable [121, 122].

##### Anxiety disorders

Alcohol is used for self-management by individuals with generalized anxiety disorder, specific phobias and panic disorders [120, 123], to alleviate direct or associated symptoms and to ameliorate suffering [124, 125]. Social anxiety disorders/social phobia (SAD) is a prevalent anxiety disorder that manifests with intense anxiety and panic in social situations already at a young age [126]. It may present in a weaker form as an adolescent personality trait, but later develop into a psychiatric disorder. Affected individuals avoid social contact to the point of complete social isolation, even when they still desire these interactions. There is an enhanced level of alcohol consumption in people with SAD [127, 128]. Alcohol was shown to attenuate the emotional impact of threatening social stimuli in patients with SAD [129]. An experimental study demonstrated that alcohol self-administration in a lab setting targeting an individual blood alcohol concentration (BAC) of 0.05% reduced social performance anxiety in adults with a DSM-IV diagnosis of social phobia. Interestingly, a placebo drink with alcohol expectancy can partially mimic this effect [130]. This not only supports the efficacy of alcohol use, but also that this effect can be conditioned to pre-determined efficacy belief. Epidemiological investigations suggested that only about 20% of the diagnosed anxiety disorder patients drink alcohol with the intention to self-manage their anxiety symptoms [123, 131], with profound differences between anxiety types [132, 133].

##### Posttraumatic stress disorder (PTSD)

Exposure to traumatic events can induce PTSD and/or depression [134, 135]. PTSD patients of all ages use alcohol to self-manage PTSD symptoms, in particular when alternative self-management tools are not available [136, 137]. Enhanced alcohol use for coping with tension and stress during a traumatic event (e.g., natural disaster) may be followed by the normalization of consumption when the event is over [138]. It may also dampen intrusive cognitive symptoms, such as the distressing recollection of an aversive event during flashbacks [139].

##### Schizophrenia

Patients with schizophrenia show a significantly higher incidence of alcohol use and abuse than people without psychiatric diagnoses [140, 141]. Thereby, the relationship between alcohol use and schizophrenia is not unidirectional, but may contain several subgroups with distinct trajectories [142]. In some patients, alcohol use precedes the first symptoms of schizophrenia [143]. However, there are also patients for whom alcohol abuse coincides with or follows the emergence of schizophrenia symptoms. This may indicate a potential use of alcohol for self-management [144] to reduce social anxiety, relieve tension and depression and to relax. It may also be used in unfavourable social circumstances, such as being homeless. Alcohol was claimed to increase pleasure, reduce dysphoria, apathy and anhedonia and facilitate sleep by a subgroup of patients with schizophrenia [120, 145, 146]. The time course of alcohol use during schizophrenia development suggests that initial use for sociability and celebrations gives way to using in order to relieve depression [147].

##### Borderline personality disorder (BPD)

The hallmarks of BPD are heightened impulsivity, mood instability, dysfunctional interpersonal relationships and suicidal behaviour. The frequently accompanying cognitive symptoms and psychotic-like ideation fluctuate at high frequency [148]. Individuals with BPD often show enhanced emotional sensitivity, affective instability, increased stress reactivity and enhanced sensitivity to social threats and rejection. BPD presents clinically at a mean age of around 18 years. Its emergence is often associated with childhood abuse or traumatic events [149]. Alcohol drinking motives in BPD patients suggest an association with negative affect coping [150, 151]. However, other studies also identified enhancement as a drinking motive [151, 152].

##### Attention deficit hyperactivity disorder (ADHD)

ADHD is a developmental psychiatric disorder which emerges already in childhood and continues into adulthood. Its hallmarks are inattention, hyperactivity and impulsivity. Numerous cognitive deficits across all attention modalities, executive functions, memory, language, social cognition and arithmetic abilities are associated with ADHD [153, 154]. Individuals with ADHD use alcohol for the self-management of enhancement, stress coping [155] and sleep problems [156]. Among individuals with ADHD symptoms, self-reported ADHD severity was associated with alcohol consumption [157]. In particular, hyperactive, impulsive and inattentive symptoms were reported to mediate alcohol use [158] in individuals with ADHD. Positive arousing expectations were reported for alcohol in ADHD individuals before commencing consumption [159]. An anticipated hangover significantly moderated this relationship in a way that only ADHD individuals with low hangover expectations showed a correlation between ADHD symptoms and alcohol intake frequency [160]. Protective behavioural strategies, which are associated with generally lower alcohol consumption, might be more effective in individuals with ADHD symptoms [161]. Overall, the view that ADHD is associated with higher alcohol consumption and that individuals with ADHD consume alcohol for self-management in other ways than normal individuals, remains controversial [154].

#### Alcohol in the self-management of pain

Individuals in adult and old age with pain conditions of various origins use alcohol to ease suffering from pain [162–164]. Patients with fibromyalgia who consumed alcohol at moderate to low levels to self-medicate showed lower levels of symptoms and a better physical quality of life than non-drinkers [165, 166]. HIV/ AIDS patients reported using alcohol to alleviate peripheral neuropathy [167]. Furthermore, alcohol is used to self-manage pain-related anxiety [168]. This use of alcohol has been confirmed in numerous experimental studies in humans [169]. However, not all types of pain are ameliorated by alcohol use. For example, headaches and migraines are triggered by alcohol rather than being effectively attenuated [170].

#### Problems with alcohol in adulthood

Although life goals change during development, the mechanisms by which an initially successful self-management through alcohol goes wrong are essentially the same as in adolescence. As alcohol consumption continues for a long period of time during adulthood, its use becomes associated with medical problems, like certain cancers, sexually transmitted and heart diseases, as well as mental health issues and psychiatric disorders, in a dose-response relationship [93]. As behaviours change during adulthood, so can the adverse effects of alcohol when self-management goes awry. It can lead to misjudgements of one’s own person, capacities and situations. Moreover, its disinhibiting properties might lead to violence or inappropriate social and sexual behaviour. It may also reduce the motor skills necessary for, e.g., driving a vehicle or operating heavy machinery. In particular, alcohol use for the self-management of psychiatric disorder symptoms is often a gateway into a psychiatric disorder – AUD comorbidity. The tong-term use of alcohol to cope with work stress may lead to chronic fatigue, resulting in a distortion of perceptual skills, reasoning disabilities, judgement, and impaired decision-making capabilities [171].

AUD development depends on the psychosocial background and social competence of an individual. Individuals from a low social background are at higher risk for AUD [172]. High-dose acute alcohol consumption may disrupt social behaviour and social cognition [173–175], and compromise effective self-management. Acute intoxication may lead to aggressive behaviour and violence [176]. High-frequency consumption and escalated dosing may result in developing a co-morbidity of SAD/social phobia with AUD [64]. Social anxiety enhances the risk of developing AUD and reduces the use of protective behavioural strategies [177]. Virtually all psychiatric disorders that manifest in adulthood are associated with enhanced risk of AUD, such as depression [178], bipolar disorders [179], anxiety disorders [180], PTSD [181], schizophrenia [182], BPD [183] and ADHD [184]. There is also high comorbidity between chronic pain, alcohol abuse and AUD [185].

### Ageing

Ageing usually describes the period of 65 years and older when most people retire from their jobs and careers to focus on their private life. Enjoyment of life is a foremost goal in this period, which includes pair relationships, friendships, and social occupations. There may also be new responsibilities emerging, such as grandchildren care, voluntary work, or care for a sick partner. However, with increasing age, general health issues become more important and require adaptations [186, 187]. Overall alcohol consumption declines in older age, as the ability to metabolize alcohol efficiently declines and also interactions with ageing-related medications emerge [188]. With ageing, there occurs a shift in the self-management use of alcohol [189, 190].

#### Social behaviour

Securing and managing social contacts also involve regular alcohol consumption in old age [191]. Thereby, alcohol consumption is embedded in social activities in a highly ritualistic way. Drinking occasions are used to maintain social contacts at home or outside the home to enjoy the company of others. Thereby, consumption patterns usually change from binge-like consumption to a more evenly distributed consumption routine [191].

#### Hedonia end enhancement

In old age, alcohol is still perceived as having pleasurable and relaxing effects [187]. Using alcohol to self-manage hedonic tone still plays a significant role, but excessive alcohol drinking at social gathertings declines [186] (Fig. 1).

#### Managing health

Most older alcohol consumers maintain their consumption during ageing [192]. Mental health is often challenged by changes in life circumstances. Even individuals with moderate consumption throughout life use alcohol as a coping instrument [193]. During ageing, alcohol consumption motives change from social reasons towards the managing of medical problems [191, 194]. Medical reasons include pain, cardiovascular diseases, sleep disturbances, and common cold, but also mental disorders, like depression and anxiety [194–196]. In old age, when sleep time decreases and difficulties falling asleep emerge, individuals report using alcohol to self-manage sleep [194].

## Adverse effects and risks of alcohol use

Although at an older age, individuals often maintain the ability to adjust their alcohol consumption according to changing instrumentalization in the self-management, there is still a risk of adverse effects. Risky alcohol consumption (>7 drinks/week) is associated with impaired instrumental activities during daily living [197]. Neurotoxic effects of high-risk alcohol consumption may add to a natural decline in brain function and further reduce cognitive performance and emotional flexibility. Escalated alcohol consumption in old age may lead to AUD development. Given the increasing number of comorbid disorders and the shrinking mental flexibility, treatment of subjects with AUD may be more difficult at this age.

## The neurobiological mechanisms of alcohol in self-management

The proximal mechanisms for the multiple instrumentalizations and use for self-management may be found in the unique pharmacological profile of alcohol and its action in the brain [3, 4]. While the neuropharmacological research into alcohol effects mainly addressed the molecular pathways into AUD, it also revealed some mechanisms underlying the controlled use of alcohol related to lifespan and some of its specific instrumentalization goals (Table 1). However, this is not completely understood and awaits further investigation.

**Table 1:** The neuropharmacological mechanisms for alcohol instrumentalization in self-management along life span (cGMP - cyclic guanosine monophosphate,5-HT – serotonin, DA - dopamine, D1 and D2 receptors – dopamine 1 and 2 receptors, DAT – dopamine transporter, GABA – γ-amino butyric acid, GLU – glutamate, LTP – long term potenmtiation, NA – noradrenaline, Nac – nucleus accumbens, NMDA – N-methyl-D-aspartate, NO - nitric oxide, PFC – prefrontal cortex, SERT – serotonin transporter).

Life span	Management goal	Altered brain function	Alcohol effects serving self-management goal	Reference
Adolescence	Social behavior	enhanced extracellular DA levels in the Nac*	alcohol increases DA activity	Philipot & Kirstein, 2004; Badanich et al., 2007
		preserved extracellular DA, 5-HT and NA in PFC*		Staiti et al., 2011
		enhanced DA receptor and DAT expression*		Teicher et al., 1995; Andersen et al., 1997; Tarazi et al. 1998
			enhanced DA response to alcohol in the Nac*	Philipot et al., 2009; Pascual et al., 2009
			GLU increase after alcohol in the Nac	Pascual et al., 2009; Carrara-Nascimenl., 2011
			potentiated GABAergic action*	Sircar & Sircar, 2006; Fleming et al., 2012; Sircar, 2017
	Negative affect coping	potentiated GABAergic action*		Sircar & Sircar, 2006; Fleming et al., 2012; Sircar, 2017
			enhanced alcohol inhibition of hippocampal LTP*	Hiller-Sturmhöfel and Swartzwelder, 2005
			enhanced alcohol inhibition of hippocampal neurogenesis*	Crews et al., 2006; Ehlers et al., 2003; Broadwater et al., 2014
			attenuated alcohol effects on GABA in the cerebellum*	Hiller-Sturmhöfel and Swartzwelder, 2005
Adulthood	Social behavior		alcohol increases DA activity	Di Chiara & Imperato, [199]
			GLU increase after alcohol	Pascual et al., 2009; Carrara-Nascimenl., 2011
	Sexual behaviour		alcohol increases DA activity	Di Chiara & Imperato, [199]
	Enhancement		alcohol increases DA activity	Di Chiara & Imperato, [199]; Kalinichenko et al., 2019, [201]
			alcohol increases 5-HT activity	Yoshimoto et al., 1992; Easton et al., 2013, 2014;
				Kalinichenko et al., 2019, [201]
	Stress coping		alcohol increases GABA release	Weiner & Valenzuela, 2006
			alcohol increases GABAergic activity	Zhu & Lovinger, 2006; Roberto et al., [198], 2004
			alcohol attenuates GLU transmission in the Nac	Pascual et al., 2009; Carrara-Nascimento et al., 2011
	Psychiatric disorders			
	Depression	disrupted sphingolipid homeostase	re-establishment of sphingolipid homeostase (partial)	Müller et al., [21]
		attenuated DA, 5-HT and NA tissue levels	alcohol increase of DA, 5-HT and NA tissue levels	Müller et al., [21]; Kalinichenko et al., 2018
			NMDA-receptor/ No-cGMP activation	Khan et al., 2021
	Bipolar disorder	enhanced GLU activity	alcohol blunts GLU activity	Zuo et al., 2007
		dysfunctional L-type calcium channels	alcohol activates internal Ca2+ stores and L-type channels	Kelm et al., 2007; Li et al., 2020
			alcohol enhances GABA_A_-receptor mediated GABA release	Kelm et al., 2007; Li et al., 2020
	ADHD	increased DAT and SERT levels		Solanto, 2002
		dysregulation of various DA and 5-HT receptors		Tripp & Wickens, 2009
		attenuated extracellular DA and 5-HT levels	alcohol increases DA and 5-HT tissue levels	Solanto, 2002
	Pain		alcohol increase brain Met-enkephalin	Mendez et al., 2010; Schulz et al., 1980; Mitchell et al., 2012
			alcohol increase β-endorphin	Lam et al., 2008; Lam and Gianoulakis, 2011
			alcohol increases NMDA-receptor activation	Mogil et al., 1993
			DA and 5-HT increase in the brain	Navratilova et al., 2016; Sommer, 2010
Ageing	Social behavior	reduced DA tissue levels in the brain*	neurochemical alterations generally suggest that	Marshall & Rosenstein, 1990; Woods & Druse, 1996
		enhanced extracellular DA and 5-HT levels in the Nac*	alcohol may loose	Yoshimoto et al., 1998
		reduced DA reuptake and metabolism in the brain*	some of its self-management capacity in ageing	Yoshimoto et al., 2001
		reduced DA D1 and D2 receptor density in the brain*		Woods et al., 1995; Tajuddin & Druse, 1996
			reduced DA and 5-HT response to alcohol*	Yoshimoto et al., 1998
	Negative affect coping	reduced 5-HT and NA tissue levels in the brain*	alcohol reduces tissue NA levels	Miguez et al., 1999; Jaatinen et al., 2013
		reduced NA response to stress*		Cizza et al., 1995; Hastings et al., 1996
		reduced GABAergic action (α4 subunit expression)*		Sarviharju et al., 2006

*cGMP* cyclic guanosine monophosphate, *5-HT* serotonin, *DA* dopamine, *D1 and D2 receptors* dopamine 1 and 2 receptors, *DAT* dopamine transporter, *GABA* γ-amino butyric acid, *GLU* glutamate, *LTP* long term potenmtiation, *NA* noradrenaline, *Nac* nucleus accumbens, *NMDA* N-methyl-D-aspartate, *NO* nitric oxide, *PFC* prefrontal cortex, *SERT* serotonin transporter.

*Compared to adulthood.

Alcohol interacts with the neurotransmission of γ-butyric acid (GABA), which is the most abundant inhibitory transmitter in the brain. Alcohol enhances the activity of GABA at the GABA_A_-receptor [9, 10]. Acute alcohol application enhances presynaptic GABA release and increases GABAergic activity [198]. Through its interaction with neocortical GABA_A_-receptors, alcohol can directly attenuate aversive memories. These effects are crucial for alcohol’s action in the dampening of natural and acquired anxiety and stress states [9] (Table 1).

Alcohol is also well-known to increase the activity of monoaminergic modulatory neurotransmitters in the mesolimbic system of the brain [92, 199]. This action may attenuate the reward threshold of the brain [18], which can, in turn, enhance the incentive value of other stimuli [200]. Alcohol increases extracellular dopamine (DA) activity in the brain [199], which was crucially linked to reinforcement learning and maintenance of alcohol consumption [10, 18, 19]. Alcohol also increases serotonergic (5-HT) activity in the brain [92, 201]. Thereby, alcohol effects on 5-HT activity depended on sex/gender and on the emotional traits of an organism, which supports the role of alcohol in personality trait self-management [201, 202]. The acute 5-HT increase, which is a crucial mechanism for the learning of alcohol seeking and consumption, may contribute to the subsequent degree of preference and the use of alcohol for self-management of negative affective states [22, 92].

While a plethora of studies have revealed now how alcohol affects brain function [9], it might be important to consider that this picture reveals mainly the alcohol interaction with a healthy brain that is not under allostatic load. However, such an organism requires relatively few self-management actions. In a brain that gives rise to very pronounced personality traits, psychiatric disorders or that is under allostatic load [203], the neuropharmacological effects of alcohol can be largely altered and even be opposite to those in healthy brains [21, 86, 201, 202] (Table 1).

## Alcohol use disorders and other alcohol-attributable harm: a public health perspective

In considerations about instrumentalizing psychoactive drugs, including alcohol, to make other, non-drug-related behaviours more efficient, the risk of use disorders is often discussed as a potential barrier [3–5]. However, from a public health point of view, AUD is only one of the health risks of alcohol use. In fact, as a recent overview of the alcohol-attributable burden of disease and mortality from the years 2000 to 2016 showed, AUD made up only 14.9% of all alcohol-attributable disability-adjusted life years and 4.9% of alcohol-attributable mortality globally [204]. Most of the disease burden of alcohol use thus is not linked directly to AUD. However, the overall alcohol-attributable burden of disease and mortality is high: approximately every 20th death or premature year of life lost globally is associated with alcohol use. Furthermore, alcohol has been one of the top 10 risk factors for mortality and burden of disease in all comparative risk assessments to date [205].

What are the potential implications for the instrumentalization of alcohol use? In short, the functionality gains of alcohol use need to be weighed against the potential risks. This weighting will crucially depend on the life cycle. Globally, alcohol is the most important risk factor for death and the burden of disease, as measured in disability-adjusted life years for people between 15 and 40 years old [204, 206]. However, we should also consider that absolute risk, and mortality and disease risk at these ages are relatively low.

How high are the absolute risks of alcohol use? Such risks depend on the level of alcohol use and the patterns of drinking, but also on the environment, the overall disease and mortality level and other risk factors [207, 208]. An absolute life-time risk of one in one-thousand has often been cited as tolerable for voluntary, i.e., self-chosen, behaviours [207, 209]. According to Shield and colleagues’ analysis of six EU countries with different drinking patterns (Estonia, Finland, Germany, Hungary, Ireland, Italy and Poland), this threshold would suggest that maximum average drinking levels for these countries should be 8–10 g/day for women and 15–20 g/day for men [206, 209]. In other words, if people use alcohol instrumentally to gain functionality, they should not exceed these limits or risk premature mortality or early burden of disease. Obviously, if people use alcohol with different patterns of drinking (e.g., alternating heavy drinking occasions with days of abstinence), the average drinking levels would be lower, as heavy drinking occasions have been linked to additional risks for injury and ischemia [210]. While these are personal decisions, they should be made based on a well-informed awareness of the risks of alcohol use. It is highly problematic that the health risks of alcohol use are currently not well known among the general population; for example, the majority of people in Europe are not aware of the link between alcohol use and cancer [211], let alone that even light drinking significantly increases risks for many cancer types, such as breast cancer [212–214]. Admittedly, these statistical significances are often driven by the large cohorts tested, and less so by the size of the effect, which may, by itself and with a single disorder, not raise too much attention. Nevertheless, information on alcohol-related truly multi-dimensional health risks other than AUD should be increased, so people can make more informed decisions about their alcohol use.

Despite accumulating scientific evidence for alcohol use in self-management, these data are mostly qualitative. Therefore, one can currently only estimate how many people systematically use alcohol for various goals. Therefore, quantitative research must be strongly encouraged to finally outline the size of the phenomenon at the population level and determine the effect sizes of alcohol’s benefits at the individual level. This may give rise to further enquiries that stratify AUD development according to different trajectories and distinct neurobiological mechanisms to best inform optimal treatment modalities.

A crucial age staring the self-management with alcohol is the adolescence. It is a period when the brain still undergoes significant structural and functional changes, which have long-lasting effects for the rest of the life [38]. Unfortunately, the demarcation of this period versus adulthood is not very well defined. As such, the picture of alcohol use for self-management we get of late adolescence and early adulthood is somewhat blurred. A more elaborated and potentially biomarker based definition of adolescence, which is aligned to the end of enhanced brain- and behavioural plasticity [215], may help here to better estimate potential benefits and long-term consequences of alcohol use.

## Self-management and the risk of alcohol abuse and addiction

The majority of humans of all ages who regularly consume alcohol for self-management, control their consumption rather well. This means their alcpohol intake is not compulsive, and its dose and frequency can still be adjusted according to the beneficial outcome and undesired effects. However, a small percentage of individuals make the transition from controlled alcohol use and instrumentalization to abuse. They develop AUD and often comorbid mental as well as physical disorders [216]. Today, it is not possible to predict who will lose control and develop addictive behavioural patterns and who is able to regain control over alcohol use. As such, even successful self-management with alcohol at a non-addictive stage has to be considered a hazardous behaviour and should never be recommended by health professionals.

## Personalized informed use

Alcohol use is based on a deeply rooted genetic inheritance that enables coping with acute toxicity and evolved together with the cultural inheritance of alcohol instrumentalization [1, 2, 5]. Even when this cultural inheritance does not achieve complete population penetrance (i.e., even abstainers may have learned the behaviour from cultural trajectories, but do not express it), it may not be easily eliminated [217]. Between prohibition and inherited use, however, there should be a third way of handling this phenomenon: personalized informed use. In this approach, personal alcohol consumption for self-management may be recognized as a non-addictive use, which is often beneficial on a highly individual base. Users maintain active control over their consumption, but also accept resulting individual physical impairments up to a certain level [218]. This would suggest that personalized risk factors, e.g., an individual genetic, epigenetic, developmental and environmental risk profile, should also be accessible at a low threshold. Psychoeducational programs in prevention and therapy should disseminate goals of alcohol instrumentalization and their risks and allow for informed decisions on alcohol use and self-identification of use patterns. This may even be actively trained in users with enhanced risk profiles for AUD. Not necessarily complete abstinence, but control over alcohol use has to be kept up under permanent consideration of alternative self-management tools, which, however, can only be elaborated once personal alcohol benefits have been recognized by a single individual.

## Conclusions

Despite its dangerous nature, accumulating evidence suggests that humans use alcohol to a large extent successfully for self-management. This is based on its easy availability in many cultures and its pharmacological profile. Alcohol has indeed features that can, when applied at a low to medium dose and frequency range, have beneficial effects on several behaviours and on cognition. This is supported by its selective neuropharmacological action, which at least partially reverses malfunctioning brain mechanisms. It may work here in the absence of other self-management tools or capabilities. However, also small quantity use of alcohol comes with adverse health effects and risks. But as this behaviour is deeply rooted in many cultures as part of their cultural inheritance it may not be easily completely abandoned. Here we suggested a personalized informed use as a potential alternative for health professional action. However, when individuals in need seek help in self-management, alcohol use should neither be introduced nor encouraged, but alternative behavioural or cognitive tools for self-management should be favoured.
